# Genome Sequence of *Pseudomonas chlororaphis* Strain PA23

**DOI:** 10.1128/genomeA.00689-14

**Published:** 2014-07-17

**Authors:** Peter C. Loewen, Jacylyn Villenueva, W. G. Dilantha Fernando, Teresa de Kievit

**Affiliations:** a Department of Microbiology, University of Manitoba, Winnipeg, Manitoba, Canada; b Department of Plant Science, University of Manitoba, Winnipeg, Manitoba, Canada

## Abstract

*Pseudomonas chlororaphis* strain PA23 is a plant-beneficial bacterium that is able to suppress disease caused by the fungal pathogen *Sclerotinia sclerotiorum* through a process known as biological control. Here we present a 7.1-Mb assembly of the PA23 genome.

## GENOME ANNOUNCEMENT

*Pseudomonas chlororaphis* strain PA23 is a soybean root isolate that is able to protect canola from stem rot disease caused by the fungal pathogen *Sclerotinia sclerotiorum* (Lib.) de Bary (1, 2). This bacterium secretes a wide range of compounds, including the antibiotics pyrrolnitrin, phenazine 1-carboxylic acid (PCA), and 2-hydroxyphenazine (2-OH-PHZ), together with chitinase, protease, lipase, and siderophores (3, 4). We have established that pyrrolnitrin is essential for PA23-mediated biocontrol (5). As is the case for many biocontrol pseudomonads, expression of PA23 antifungal factors is governed by a complex regulatory hierarchy. One of the key elements is the GacS/GacA two-component signal transduction system, which works in concert with the Rsm system (4, 6). Additional regulators that oversee production of antifungal compounds include the PhzI/PhzR quorum-sensing system (7), the stationary-phase sigma factor RpoS (8), a transcriptional regulator of RpoS known as PsrA (6), and a global stress response called the stringent response (8).

The genome of *P. chlororaphis* PA23 was sequenced utilizing a Pacific Biosciences data set generated by GenomeQuebec, which was assembled using the PacBio SMRT Analysis pipeline version 2.2.0 (http://www.pacificbiosciences.com) with 72-fold coverage to give a single contiguous genome sequence. The sequence was annotated by the NCBI prokaryotic genomes annotation pipeline.

The *P. chlororaphis* PA23 genome consists of 7,122,173 bases with a G+C content of 62.6%. There are 6,179 putative coding sequences, 68 tRNA genes, and 5 rRNA clusters. In addition, biosynthetic loci for pyrrolnitrin, phenazine, hydrogen cyanide, and alkaline protease have been identified, which is consistent with exoproducts secreted by this bacterium. Comparison of the genome with those of the two other completed *P. chlororaphis* genomes, O6 (CM001490) and subsp. *aureofaciens* 30-84 (CM001559) (9), using Mauve version 2.3.1 (10) revealed a ~600 kbp inversion compared to both O6 and 30-84, together with a short 60-kbp rearrangement relative to O6 alone.

### Nucleotide sequence accession number.

The genome sequence of *P. chlororaphis* PA23 has been deposited at the NCBI GenBank under the accession number CP008696.
